# Correlation between hydrogen production rate, current, and electrode overpotential in a solid oxide electrolysis cell with La_0.6_Sr_0.4_FeO_3−*δ*_ thin-film cathode

**DOI:** 10.1007/s00706-014-1220-y

**Published:** 2014-05-24

**Authors:** Gregor Walch, Alexander Karl Opitz, Sandra Kogler, Jürgen Fleig

**Affiliations:** Institute of Chemical Technologies and Analytics, Vienna University of Technology, Vienna, Austria

**Keywords:** Electrochemistry, Oxides, Electron transfer, Cathode, Overpotential

## Abstract

**Abstract:**

A solid oxide electrolysis cell (SOEC) with a model-type La_0.6_Sr_0.4_FeO_3−*δ*_ thin-film cathode (working electrode) on an yttria-stabilized zirconia electrolyte and a porous La_0.6_Sr_0.4_Co_0.2_Fe_0.8_O_3−*δ*_ counterelectrode was operated in wet argon gas at the cathode. The hydrogen formation rate in the cathode compartment was quantified by mass spectrometry. Determination of the current as well as outlet gas composition revealed the electrochemical reduction of some residual oxygen in the cathodic compartment. Quantitative correlation between gas composition changes and current flow was possible. At 640 °C a water-to-hydrogen conversion rate of ca. 4 % was found at −1.5 V versus a reversible counterelectrode in 1 % oxygen. Onset of hydrogen formation could already be detected at voltages as low as −0.3 V. This reflects a fundamental difference between steam electrolysis and electrolysis of liquid water: substantial hydrogen production in a SOEC is already possible at pressures much below ambient. This causes difficulties in determining the cathodic overpotential of such a cell.

**Graphical Abstract:**

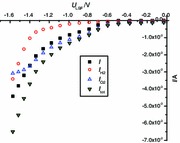

## Introduction

Temporal and spatial variations of the power output from sustainable energy sources such as wind and solar energy urgently require efficient energy storage systems. Chemical storage by electrolysis of water is highly promising in this respect. Water electrolysis may also play a key role in a future hydrogen economy with electric cars based on fuel cells. Electrolysis cells can operate with different electrolytes and at temperatures ranging from typically 70 to 1000 °C. The theoretical efficiency of a water electrolysis cell is above 100 %—if surplus thermal energy is supplied from the surroundings—and increases further with increasing temperature. Also the kinetics of electrochemical processes generally improve with increasing temperature. Both facts make high-temperature electrolysis cells very attractive, and solid oxide electrolysis cells (SOECs), which can be operated at temperatures above ca. 600 °C, have therefore come into the focus of research [1].

Most SOEC systems investigated to date have been solid oxide fuel cells (SOFCs) operating in reverse mode [2–13]. The cathode of such SOECs consists of a Ni/yttria-stabilized zirconia (YSZ) composite, and the air-exposed anode is based on a perovskite-type oxide, e.g., Sr-doped LaMnO_3_ (LSM) or LaCo_*x*_Fe_1−*x*_O_3−*δ*_ (LSCF). However, the reverse overpotentials at the electrodes of SOECs cause different electrode performance compared with SOFCs and partly higher degradation rates [1, 5, 14–17]. Hence, optimization of the electrodes is one of the main issues in current SOEC research.

To gain in-depth understanding of the kinetics on promising SOFC and SOEC electrode materials, model-type thin-film microelectrodes can be employed [18]. Recently, a novel microelectrode design was developed that unravels details of the reaction mechanism of SrTi_1−*x*_Fe_*x*_O_3−*δ*_ and La_1−*x*_Sr_*x*_FeO_3−*δ*_ (LSF) thin-film electrodes [19]. However, testing thin-film electrodes under more realistic SOEC operating conditions is also of relevance in this context; in particular, detection of the produced hydrogen can complement microelectrode investigations.

In this work, we therefore employed model-type La_0.6_Sr_0.4_FeO_3−*δ*_ thin-film electrodes with a thin-film current collector grid as the water-splitting cathode on YSZ solid electrolytes. The current–voltage characteristic was measured for SOECs with porous LSCF counterelectrodes in oxygen. Different contributions to the overall cell voltage were separated. Additionally, in contrast to many other SOEC studies, we did not expose the cathode to a H_2_O/H_2_ gas mixture and could thus investigate the “threshold” voltage for hydrogen production using mass spectrometry. The measured currents are correlated with the hydrogen formation rate, and the measurability of cathodic overpotentials in such experiments is discussed.

## Results and discussion

### Impedance spectroscopy measurements

The voltage applied between the working electrode (WE) and counterelectrode (CE) of an electrolysis cell *U*_WE–CE_ (<0 in electrolysis mode) includes the thermodynamically required voltage for water splitting *U*_td_ (<0), the voltage drop at the ohmic electrolyte resistance (*η*_Ω_ > 0), and the overpotentials *η* of the WE and CE (*η*_WE_ < 0, *η*_CE_ > 0). The cell voltage is therefore given by

1 $$U_{{{\text{WE}}{-}{\text{CE}}}} = U_{\text{td}} + \eta_{\text{WE}} - \eta_{\text{CE}} - \eta_{\varOmega }$$

or

2 $$\left| {U_{{{\text{WE}}{-}{\text{CE}}}} } \right| = \left| {U_{\text{td}} } \right| + \left( {\left| {\eta_{\text{WE}} } \right| + \eta_{\text{CE}} + \eta_{\varOmega } } \right).$$

Equation (2) better reflects the fact that the applied voltage includes a thermodynamically required contribution (*U*_td_) and contributions related to the kinetic and transport properties of the electrodes and electrolyte.

**Fig. 1:**
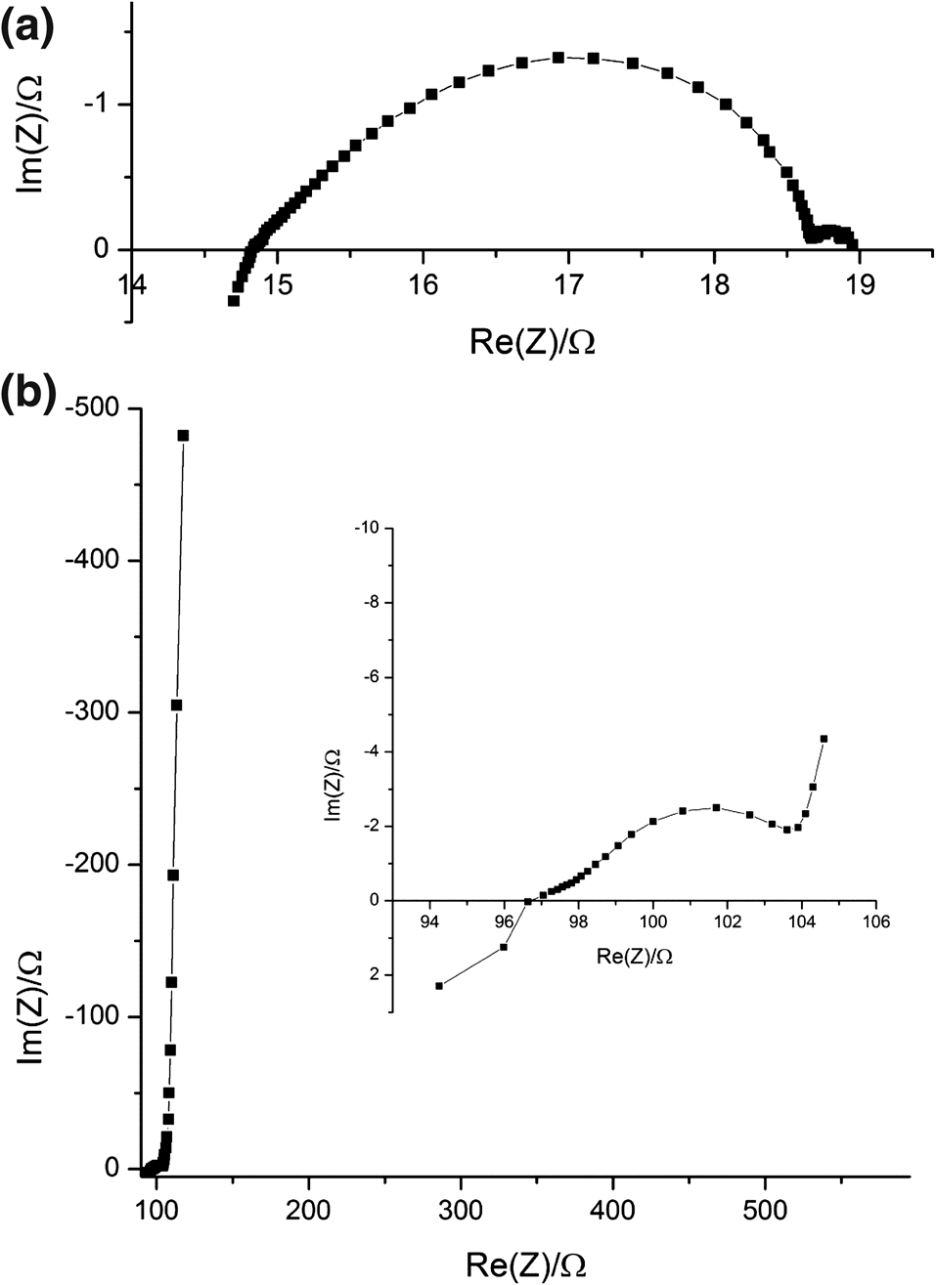
**a** Impedance spectrum of a symmetrical LSCF/YSZ/LSCF cell in 1 % oxygen (N_2_ carrier gas) measured at 649 °C. The size of the arc indicates the polarization resistance of the two LSCF electrodes. **b** Impedance spectrum of a SOEC (LSF/YSZ/LSCF) measured at 640 °C in wet argon at the cathode and 1 % oxygen (N_2_ carrier gas) at the anode under short-circuit conditions. The high-frequency intercept (see inset) was used to determine the ohmic polarization in subsequent current–voltage measurements on the same cell

For detailed characterization of the LSF electrode under load, knowledge of the counterelectrode and ohmic polarization is required. Therefore, two impedance measurements were performed. Firstly, a symmetrical cell with porous LSCF electrodes on both sides was investigated in 1 % O_2_ (the gas used at the anode side in the following SOEC experiments). The corresponding impedance spectrum is shown in Fig. 1a. The high-frequency intercept reflects the electrolyte resistance, and the distorted arc represents the polarization of the two LSCF electrodes. A polarization resistance of ca. 2 Ω per electrode results at ~649 °C, indicating acceptable electrode quality. Electrodes of the same type and size were also used in the SOFC experiments, and their resistance value is therefore orders of magnitude smaller than the polarization resistances measured for our LSF thin-film working electrodes in wet nitrogen or in hydrogen-containing atmosphere. Hence, the current–voltage curves found in this study are not significantly affected by the polarization of the LSCF counterelectrode (*η*_CE_ ≈ 0).

Secondly, the ohmic YSZ electrolyte resistance was obtained from an impedance spectrum measured on a SOEC with LSF (thin film) working electrode and LSCF counterelectrode under short-circuit conditions. The corresponding impedance spectrum is shown in Fig. 1b and indicates an ohmic resistance *R*_ion_ (high-frequency intercept) of about 97 Ω at 640 °C. The reason for obtaining a much larger resistance value compared with the symmetrical cell (Fig. 1a) is simply the different geometry of the LSCF and LSF electrodes, see “Experimental”, and the different electrolyte thickness. In the following dc experiments, this resistance (*R*_ion_) was used to calculate the ohmic overpotential *η*_Ω_ of the operating SOEC from the measured current |*I*| via *η*_Ω_ = *R*_ion_·|*I*|.

### Current–voltage curves and gas analysis

**Fig. 2:**
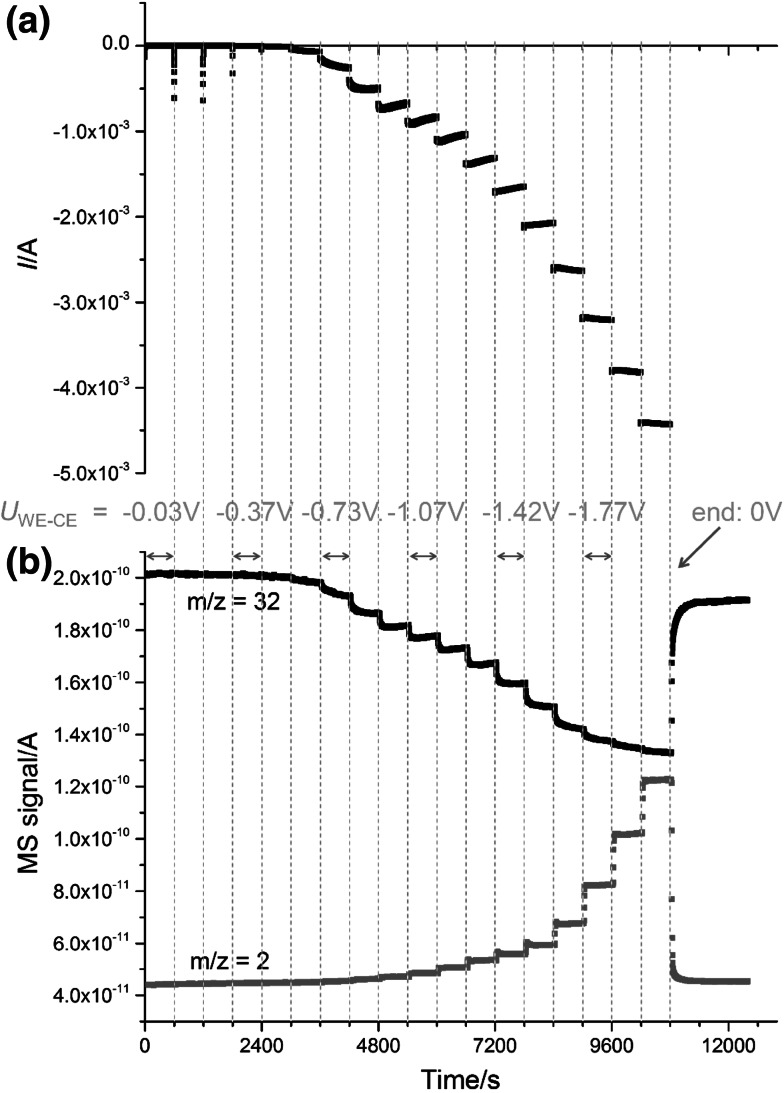
**a** Current versus time measured for cell voltages *U*_WE–CE_ from 0 to −2.0 V. Some cell voltages are indicated. **b** Mass spectrometer signals corresponding to hydrogen (*m*/*z* = 2) and oxygen (*m*/*z* = 32) for the same measurements, i.e., for changing cell voltage

Figure 2a displays the current measured in the SOEC when applying cell voltages *U*_WE–CE_ between 0 and −2.0 V. The time dependence of the current for a given voltage is partly due to the charging of the electrode capacitances and possibly also due to some slight irreversible changes upon polarization. However, sufficiently stable currents are found for all voltages after several tens of seconds, and a current–voltage curve can be determined from these data (see below).

Figure 2b shows the signals from the mass spectrometer (connected to the cathode compartment of the sample holder) for *m*/*z* = 2 (hydrogen) and 32 (oxygen) for the same measurement. It can be seen that the hydrogen signal increases with increasing current in the SOEC. For the highest electrochemical current, normalization of the MS-signal indicates that the working electrode outlet gas contains 0.11 % hydrogen. Taking the initial water content of 2.6 % into account, this corresponds to a water-to-hydrogen conversion rate of 4.2 %. Moreover, hydrogen is already detected for cell voltages much smaller than the voltage of water decomposition for standard conditions, which is −1.02 V at 640 °C. This is discussed in more detail below.

The mass spectrum also shows that some residual oxygen is present in the H_2_O/Ar gas stream on the working electrode side—normalization reveals a concentration without current of ca. 0.11 %. This concentration was found to depend on the oxygen content in the counterelectrode gas compartment, suggesting a small leak between the counterelectrode and working electrode compartments [20]. Upon application of a cathodic voltage, the oxygen content decreases, clearly indicating that oxygen is pumped electrochemically to the other side of the SOEC cell.

Since *η*_CE_ can be neglected in our case and the ohmic overpotential can be determined from the dc current and ionic resistance (see above), we can plot the steady-state currents from Fig. 2a versus the voltage of the LSF electrode *U*_LSF_ defined by

3 $$U_{\text{LSF}} = U_{{{\text{WE}}{-}{\text{CE}}}} + \eta_{\varOmega } ( + \eta_{\text{CE}} ) = U_{\text{td}} + \eta_{\text{WE}} .$$

**Fig. 3:**
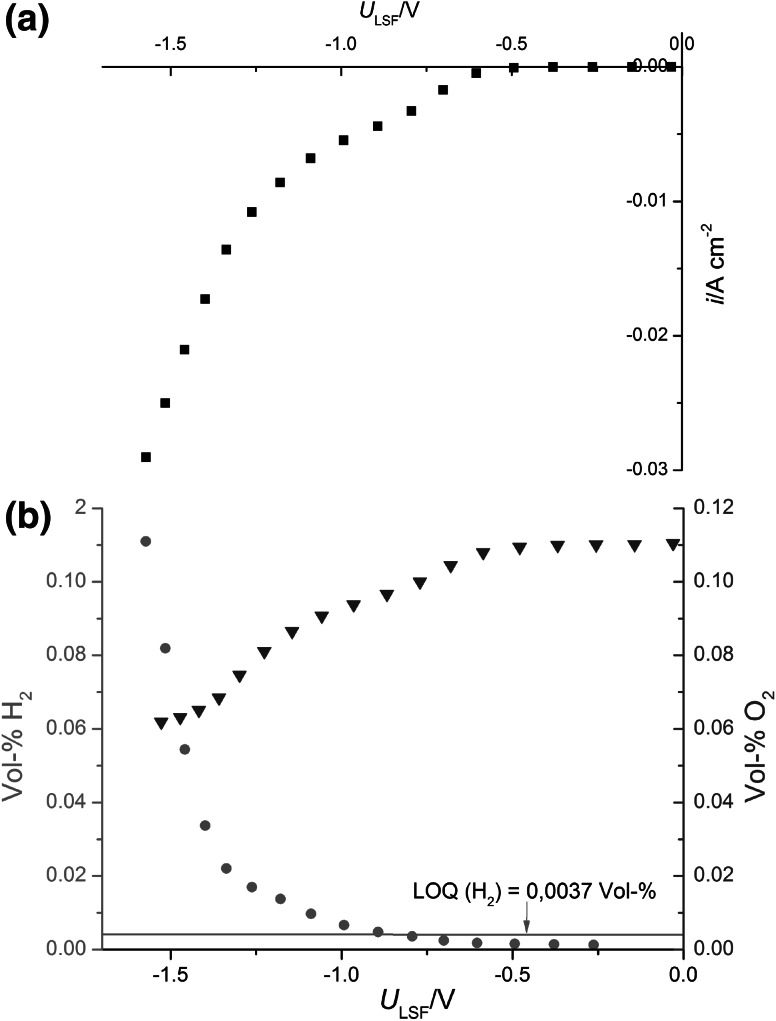
**a** Current density–voltage curve determined from the steady-state values in Fig. 2a; the current density (*i*) is referred to the free LSF surface of the WE. *U*_LSF_ was calculated from *U*_WE–CE_ according to Eq. (3) with *η*_CE_ = 0. **b** Hydrogen and oxygen gas concentrations for different voltages *U*_LSF_ calculated from the mass spectrometer signals in Fig. 2b. LOQ indicates the limit of quantification (concentration corresponding to blank value plus ten times the standard deviation of the blank value)

The voltage *U*_LSF_ corresponds to the ohmic-drop-corrected voltage of a polarized LSF electrode versus a reversible counterelectrode in 1 % oxygen and is negative in electrolysis mode. Figure 3a displays the resulting current–voltage curve. Here, current densities are plotted, calculated with respect to the free surface area of the LSF working electrode. The nonlinearity of the curve may have several causes, such as a rate-limiting charge-transfer reaction or stoichiometry polarization of the electrode. However, a mechanistic interpretation would require many more measurements and lies beyond the scope of this paper.

For each value of *U*_LSF_ the measured signals of the mass spectrometer can be transferred into concentrations. This results in Fig. 3b, which indicates decreasing oxygen content for *U*_LSF_ voltages more negative than ca. −0.4 V; for the most negative voltage of ca. −1.5 V, a decrease to almost half of the original value occurred. As already concluded from Fig. 2b, significant amounts of hydrogen were produced for the most negative voltages, but hydrogen can already be detected for *U*_LSF_ values as small as −0.3 V. This is caused by a fundamental difference between high-temperature steam electrolysis and electrolysis in aqueous electrochemistry (see below).

**Fig. 4:**
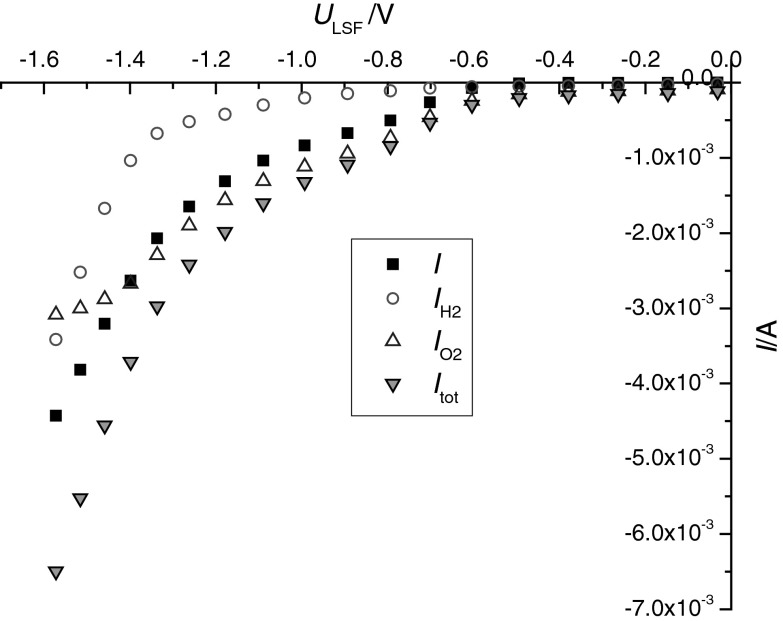
Current–voltage curves calculated from the detected changes of hydrogen ($I_{{{\text{H}}_{ 2} }}$) and oxygen concentrations ($I_{{{\text{O}}_{ 2} }}$) in the working electrode gas flow. The sum curve (*I*_tot_) of these two calculated currents corresponds acceptably well to the measured dc currents (*I*)

For known gas flow rates, the gas concentrations shown in Fig. 3b can be transferred into currents via Faraday’s law. Figure 4 displays the calculated currents reflecting oxygen removal from the counterelectrode compartment (*I*_O2_) and hydrogen production therein (*I*_H2_). The oxygen current clearly dominates for lower voltages, which is not surprising since the thermodynamic voltage *U*_td,O2_ to be overcome by *U*_LSF_ is small, being given by

4 $$U_{{{\text{td,O}}_{ 2} }} = \frac{RT}{4F}{ \ln }\frac{{p}_{\text{O}_{{ 2 ,\,{\text{WE}}}} }}{{p}_{\text{O}_{{ 2 ,\,{\text{CE}}}} }},$$

where *R*, *T*, and *F* denote the gas constant, temperature, and Faraday’s constant, respectively. For oxygen partial pressures in the two gas flows of ${{p}_{\text{O}_{{ 2 ,\,{\text{CE}}}} }}$ = 1 % and ${{p}_{\text{O}_{{ 2 ,\,{\text{WE}}}} }},$ = 0.11 % (normalized to 1 bar), this corresponds to −45 mV.

For very negative voltages, however, the current due to hydrogen production becomes similar to that due to oxygen removal. The sum of the two currents (*I*_tot,calc_), calculated from the H_2_ and O_2_ concentrations in the outlet gas, should correspond to the electrically measured current (*I*_meas_). Indeed, the measured and calculated currents are in reasonable agreement, demonstrating the validity of the calculations and the absence of substantial additional sources of dc current in our cell. The fact that a nonzero current is calculated for oxygen production at *U*_LSF_ = 0 is simply an artifact caused by MS signal drift.

### The threshold voltage in SOECs and consequences for overpotential definition

In liquid water, the H_2_O activity is unity and by electrolysis large amounts of hydrogen as well as oxygen are only produced when gas bubbles can develop, i.e., when oxygen and hydrogen have chemical potentials that correspond to the ambient pressure. Therefore, at room temperature and 1 bar ambient pressure, the voltage corresponding to the standard Gibbs free energy of reaction (−1.23 V) is the thermodynamic threshold for electrolytic water splitting. For lower voltages, the surface coverage of adsorbed species changes and small amounts of hydrogen and oxygen dissolve in the water, but substantial gas production does not take place [21].

In SOECs, however, gaseous water is used and the partial pressure of all gases is variable. The thermodynamic voltage of water decomposition is given by

5 $$U_{\text{td}} = U_{\text{td}}^{0} + \frac{RT}{2F}{ \ln }\frac{{p_{{\text{H}}_{ 2} {\text{O}}}}}{{\sqrt {p_{{\text{O}}_{2}} } \cdot p_{{\text{H}}_{ 2} }}},$$

with *p*_*i*_ being the partial pressure of species *i*, normalized to 1 bar. $U_{\text{td}}^{0}$ (<0) is the decomposition voltage for standard conditions (*p*_*i*_ = 1). Even for known $p_{{\text{H}}_{ 2} {\text{O}}}$ (at the WE) and $p_{{\text{O}}_{ 2}}$ (at the CE) it is not possible to specify a decomposition voltage of water. Supposing that removal of the produced hydrogen is very fast (i.e., $p_{{\text{H}}_{ 2}}$ stays at very low values), a substantial electrolysis current is possible already for |*U*_WE–CE_| far below |$U_{\text{td}}^{0}$| and a “threshold” for onset of substantial hydrogen production does not exist; for example, an outlet gas flow at the working electrode of 10 dm^3^/min (measured at room temperature) with 10 ppm electrolytically produced H_2_ corresponds to a Faradaic current of ca. 13 mA at a *U*_td_ value of −535 mV (calculated for $p_{{\text{H}}_{ 2} {\text{O}}}$ = 1 bar and $p_{{\text{O}}_{ 2}}$ = 0.2 bar) and thus a voltage much below the standard decomposition voltage of water (−1020 mV at 640 °C). In our specific experiments, hydrogen production could already be observed for *U*_LSF_ = −300 mV.

This also leads to an inherent problem when aiming to separate *U*_LSF_ into its two terms, cf. Eqs. (3) and (5): when varying *U*_LSF_, the Faradaic current and thus also $p_{{\text{H}}_{ 2}}$ in the gas stream varies. Accordingly, a cell with given $p_{{\text{O}}_{ 2}}$, $p_{{\text{H}}_{ 2} {\text{O}}}$, *T*, and gas flow rates still does not have a well-defined *U*_td_ value—it depends on the current. Therefore, a variation of the voltage *U*_LSF_ (and thus *I*) varies both *U*_td_ and *η*_WE_, and determination of *η*_WE_ is difficult. One may regard separation of *U*_LSF_ into a thermodynamic part (*U*_td_) and a kinetic part (*η*_WE_) as somewhat arbitrary, particularly since also voltage contributions due to diffusion limitation in the electrode can be described by Nernst’s equation, analogous to Eq. (5). However, for a given current, the $p_{{\text{H}}_{ 2}}$ in the gas stream does not depend on any electrochemical or geometrical parameters of the working electrode. Therefore, we consider separation of the electrode-independent voltage part *U*_td_ according to Eq. (5) to be meaningful, with *p*H_2_ reflecting the electrolytically produced hydrogen partial pressure outside the electrode. Voltage contributions due to diffusion-related concentration changes of H_2_ within a porous electrode, however, are still attributed to *η*_WE_.

In principle, a reference electrode exposed to the same *p*H_2_ as the cathodic working electrode could reveal the kinetic overpotential *η*_WE_. However, then it has to be ensured that the reference electrode is exposed to the gas produced by the working electrode (i.e. to the outlet gas stream rather than to the inlet gas stream). Alternatively, gas analysis of the outlet gas and Eq. (5) may be used to calculate *U*_td_. Unfortunately, in our specific experiments, the situation is further complicated by the fact that also residual oxygen is present in the working electrode gas compartment and thus a mixed potential is established. Hence, calculation of *U*_td_ was not possible, despite knowing *p*H_2_.

Finally, it should be emphasized that such a problem in determining the working electrode overpotential does not exist when investigating SOECs with defined inlet H_2_O/H_2_ gas mixtures and sufficiently high flow rates, i.e. the case for which the electrolytically produced hydrogen does not significantly change *p*H_2_. Also in aqueous solutions this is not an issue since, for fixed ambient pressure and substantial hydrogen production, *p*H_2_ is fixed to 1 bar. The voltage regime with very small current due to dissolution of H_2_ in water would also face the problems discussed above but is of minor importance in aqueous electrochemistry.

## Conclusions

A SOEC using a LSF model-type thin-film working electrode could be successfully operated. The produced hydrogen could be quantified by a mass spectrometer. Up to ca. 4 % of the water in the gas stream at the working electrode was electrolytically split at 640 °C. Residual oxygen and its electrochemical removal from the working electrode compartment by the applied voltage could also be quantified. The calculated Faradaic currents of O_2_ removal and H_2_ production corresponded acceptably well to the experimentally measured electrical current. The onset of detectable hydrogen production takes place at voltages much smaller than the decomposition voltage of water for standard conditions—here at voltages as small as −0.3 V versus a reversible electrode at 10 mbar O_2_. This is caused by a fundamental difference between electrolysis of liquid water and steam electrolysis, since in the latter case and thus also in SOECs large amounts of hydrogen can evolve with pressures much below ambient. This, however, causes inherent problems when determining the overpotential of such working electrodes in SOECs.

## Experimental

**Fig. 5:**
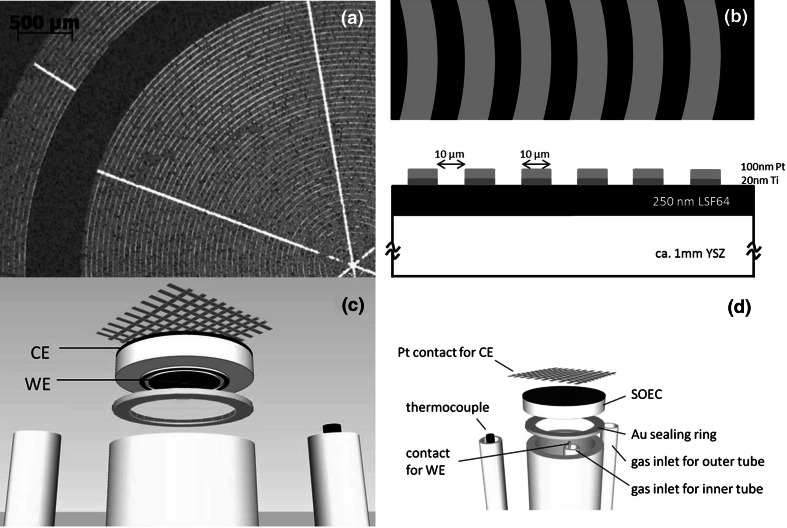
**a** Bright-field image of the circular LSF thin-film electrode on YSZ with the ring-shaped Pt current collector pattern (Pt appears bright, rings with 10 µm width). The white lines are broad Pt current-collecting strips (30 µm width). The separated outer Pt rings were not used in this study. **b** Sketch of top view and cross-section of a part of the working electrode illustrating the geometry and position of the oxide thin film and the Pt grid. **c**, **d** Sketch of the entire cell setup (*WE*: LSF working electrode, *CE*: porous counterelectrode)

Model-type La_0.6_Sr_0.4_FeO_3−*δ*_ (LSF) thin-film electrodes of 250 nm thickness were prepared by pulsed laser deposition (KrF excimer laser: Lambda COMPex Pro 201F, wavelength 248 nm) at substrate temperature of 610 °C and oxygen partial pressure of 0.04 mbar. As an electrolyte, disk-shaped YSZ polycrystals (10 mm diameter, 1–2 mm thick) were made from 8 mol % Y_2_O_3_-doped zirconia powder (Tosoh, Japan) by pressing and sintering at 1550 °C for 5 h. The electrical conductivity of *p*-type LSF strongly decreases with decreasing oxygen partial pressure due to changing charge compensation of the dopant [22, 23], and thus substantial electrical sheet resistance may occur in LSF thin-film electrodes when operated in H_2_-containing atmospheres. Therefore, ring-shaped Pt/Ti current collectors were deposited on the LSF thin film by sputtering and photolithography (Ti: 20 nm thickness, Pt: 100 nm thickness). Figure 5a, b display an image of the resulting working electrode (5 mm diameter) and a sketch of the LSF film with the current collector geometry, respectively. These electrodes resemble those used in studies on ceria anodes for SOFCs [24]. The outer Pt ring seen in Fig. 5a is disconnected from the remaining part of the current collector. It might be employed as a reference electrode but was not used in this study.

Details of the LSF film preparation and structural characterization, the electrochemical properties of such LSF thin-film electrodes with Pt collectors on top or beneath, and the role of the electrical sheet resistance are reported in a separate paper. There, it is also shown that the bulk path of the hydrogen evolution/oxidation reaction is predominant in this type of electrode, meaning that large parts of the oxide surface contribute to the electrochemical reaction and oxide ions are transported through the electrode.

A counterelectrode of porous La_0.6_Sr_0.4_Co_0.2_Fe_0.8_O_3−*δ*_ (LSCF) was deposited via a slurry on the opposite side of the YSZ electrolyte. For quality tests of the LSCF counterelectrodes, also symmetrical cells with two porous LSCF electrodes on a YSZ polycrystal were prepared. The symmetrical LSCF/YSZ/LSCF cells were investigated at ~649 °C by impedance spectroscopy in 1 % oxygen in N_2_ (on both sides) using a Solartron SI 1260 impedance gain/phase analyzer (frequency range 1 MHz–50 mHz, 0.01 V_rms_, four-wire mode). For full cell tests, the SOEC with LSF thin-film cathode and porous LSCF anode was mounted in a Probostat sample holder (NorECS, Norway) and exposed to two different gas atmospheres (Fig. 5c, d). Wet argon gas was used at the LSF thin-film working electrode (2.6 % H_2_O, flow rate ca. 24 cm^3^/min) and 1 % O_2_ in N_2_ at the LSCF counterelectrode (flow rate ca. 22 cm^3^/min). The water content of the working electrode gas was established by bubbling through deionized water at room temperature. The reason for not using ambient air at the counterelectrode was a small leak in the cell, leading to some residual oxygen in the cathodic compartment; this residual oxygen level could be decreased by using 1 % instead of 20 % oxygen in the counterelectrode compartment. Impedance measurements on the short-circuited full cell (Alpha-A high-performance frequency analyzer with electrochemical test station Pot/Gal, Novocontrol, Germany, frequency range 1 MHz–50 mHz, 0.01 V_rms_) allowed determination of the electrolyte resistance at 640 °C.

Dc measurements between the two electrodes of the SOEC were performed with a Keithley 2611A source–measure unit at 640 °C with voltages *U*_WE–CE_ from 0 to −2.0 V and acquisition time of 600 s per set voltage. The gas composition of the outlet gas from the cathode compartment was continuously analyzed by a mass spectrometer (Pfeiffer OmniStar gas analysis system GSD320 containing a QMG220 PrismaPlus compact mass spectrometer; EI-Q-MS, quartz sample inlet capillary). Calibration of the mass spectrometer was done using gas mixtures with known fraction of H_2_ or O_2_ bubbled through a gas washing bottle and recording the signals for mass-to-charge ratios of *m*/*z* = 2 and 32 for H_2_ and O_2_, respectively. From the calculated gas concentrations and the known gas flow rates, the hydrogen production rate upon current flow as well as the rate of oxygen consumption could be calculated.
